# Association between human immunodeficiency virus serostatus and the prevalence of atrial fibrillation

**DOI:** 10.1097/MD.0000000000026663

**Published:** 2021-07-23

**Authors:** Ngozi Osuji, Sabina A. Haberlen, Hiroshi Ashikaga, Todd T. Brown, Matthew J. Feinstein, Mallory D. Witt, Jared W. Magnani, Elsayed Z. Soliman, Katherine C. Wu, Wendy S. Post

**Affiliations:** a Johns Hopkins University Bloomberg School of Public Health, Baltimore, MD; b Department of Medicine, Johns Hopkins University School of Medicine, Baltimore, MD; c Department of Medicine, Northwestern University Feinberg School of Medicine, Chicago, IL; d The Lundquist Institute at Harbor-University of California Los Angeles, Torrance, CA; e Department of Medicine, University of Pittsburgh, PA; f Wake Forest School of Medicine, Winston-Salem, NC.

**Keywords:** arrhythmia, atrial fibrillation, atrial flutter, heart diseases, human immunodeficiency virus

## Abstract

Atrial fibrillation (AF) leads to increased risk for stroke. Human immunodeficiency virus (HIV) is associated with cardiovascular disease (CVD), although it is unclear if HIV is associated with AF. The purpose of this study was to evaluate the association between HIV serostatus and the prevalence of AF in the Multicenter AIDS Cohort Study.

A cross sectional study was conducted among 1674 HIV-infected (HIV+) and uninfected (HIV–) men who completed resting 12-lead electrocardiograms, and/or ambulatory electrocardiogram monitoring. Multivariable logistic regression was used to evaluate the association between AF, defined as the presence of either AF or atrial flutter, and HIV+ serostatus. Associations were adjusted for demographic variables, and then also for CVD risk factors.

HIV+ men were younger than HIV– men (median 55.5 vs 61.7 years, *P* < .001) and were more frequently African-American (30.5% vs 17.8%, *P* < .001). Most HIV+ men (81%) had undetectable viral load. The age and race adjusted prevalence of AF was 3.0% in HIV+ and 3.3% in HIV– men. There was only 1 case of AF among African-American men. There were no associations between AF and HIV serostatus after adjusting for demographic factors (odds ratio 0.76; 95% CI 0.37 to –1.58; *P* = .47) or after further adjustment for CVD risk factors (odds ratio 0.84; 95% CI 0.39 to –1.81; *P* = .66).

We found no association between HIV and AF in this cohort in which viral replication among the HIV+ men is generally suppressed. The overall prevalence of AF was low and was rare in African-American men.

## 1 Introduction

The use of combination antiretroviral therapy (cART) has extended the life expectancy of people living with human immunodeficiency virus (HIV) infection (HIV+),^[1–3]^ which has contributed to an increase in cardiovascular disease (CVD) in the HIV+ population as the population ages.^[4]^ CVD events, such as myocardial infarction, heart failure, sudden cardiac death, and stroke are more common in HIV+ compared with HIV– individuals.^[5–9]^

The relationship between HIV infection and atrial fibrillation (AF) is unclear.^[10]^ A study from the Veterans Affairs (VA) HIV Clinical Case Registry found that low CD4+ T cell count and elevated HIV ribonucleic acid viral load were associated with incident AF.^[11]^ A more recent matched case-control study in the HIV Electronic Comprehensive Cohort of CVD Complications found an elevated unadjusted odds of AF among HIV+ compared with HIV– individuals, which was not significant after adjustment for demographics.^[12]^ Data from the Healthcare Cost and Utilization project in California found that HIV was associated with incident AF.^[13]^ Further understanding of the relationship between HIV and AF is important from a public health perspective.

The aim of our study was to examine the frequency of AF using resting 12-lead and ambulatory electrocardiographic (ECG) monitoring among a cohort of men with predominantly suppressed viral replication and ascertain whether the frequency differs by HIV serostatus.

## 2 Methods

This cross-sectional study was nested within the Multicenter AIDS Cohort Study (MACS), an ongoing prospective study of the natural and treated histories of HIV-1 infection among men who have sex with men.^[14]^ The study is conducted in 4 communities in the United States: Baltimore, MD and Washington, D.C.; Los Angeles, CA; Pittsburgh, PA; and Chicago, IL. Biological and physiological data are collected every 6 months from approximately 3000 active participants.

All active MACS participants were offered resting 12-lead ECG and ambulatory ECG examinations at their routine study visits from October 2016 to September 2017.^[15]^ Continuous ambulatory ECG monitoring was performed with a non-invasive adhesive wireless single-lead device (ZioXT, iRhythm Technologies, Inc., San Francisco, CA) placed on the participant's chest by trained research personnel. The device was worn for up to 14 days and then mailed to iRhythm in a prepaid envelope. iRhythm provided detailed individual-level data, including the presence of AF or atrial flutter, as well as a preliminary clinical report for each participant with representative ECG tracings interpreted and verified by the MACS ZioXT reading center at Johns Hopkins University. AF burden was defined as the percentage of the analyzable time in AF, ranging from 0% to 100%. Participants with <24 hours of analyzable time and/or <25% analyzable time (relative to wear time) were excluded from the analysis. The resting 12-lead ECG measurements were recorded digitally at 10 mm/mV calibration at a speed of 25 mm/s for 10 seconds using GEMSIT MAC 1600 ECG machines (Marquette Electronics, Milwaukee, WI) by trained research staff. ECG recordings were transmitted to the Epicare ECG Reading Center at the Epidemiological Cardiology Research Center of the Wake Forest School of Medicine, for centralized reading. Readers were masked to the HIV serostatus of participants.

Data were also obtained from routine semi-annual study visit records of demographics, clinical parameters, and cardiovascular risk factors. This study was approved by the Johns Hopkins institutional review board through a reliance agreement with each study site (IRB00219740). Written informed consent was obtained from the participants.

The main exposure was HIV serostatus. Serostatus was measured prospectively by an enzyme-linked immunosorbent assay; incident seroconversion was confirmed by Western blot. We compared the distribution of characteristics between groups using the analysis of variance for normally distributed variables, the Wilcoxon rank-sum test for skewed data and the Pearson chi-squared test for categorical variables. The primary outcome of interest was the presence of AF, defined as the presence of either AF or atrial flutter on the ambulatory ECG monitor and/or the resting 12 lead ECG.^[16]^ The prevalence of AF was adjusted for age (5-year increments except for youngest [18–30 years] and oldest [76–91 years] age groups) and race (Caucasian vs non-Caucasian) using direct standardization and the HIV-sample as the reference population.

We used a logistic regression model to ascertain the odds ratio of AF by HIV serostatus and other risk factors. The uneven distribution of outcomes among the Caucasian, African American and Hispanic or other racial groups, necessitated stratification into Caucasian and non-Caucasian groups to enable adjustment for race. We analyzed the following models; model 1: unadjusted; model 2: adjusted for age and race; model 3: additionally, adjusted for body mass index, cumulative pack year of smoking, use of medications to treat hypertension or diabetes, heavy alcohol use (>13 drinks/wk), fasting blood glucose, and systolic blood pressure. The 2-sided test of statistical significance was defined as a *P*-value < .05. Analysis was performed using Stata version 15.1 (College Station, Texas, USA: StataCorp LLC).

## 3 Results

The analytical sample included 1674 individuals, with 780 (47%) HIV– and 894 (53%) HIV+ participants. Baseline characteristics by HIV serostatus are shown in Table 1. The HIV+ men were younger and more likely to be African American or Hispanic/other race than the HIV– men. There were also differences in several cardiovascular risk factors. Most (81%) HIV+ men had an undetectable HIV ribonucleic acid viral load (<20 copies/mL), were on cART (91%), and HIV+ men had a median duration of cART use of 12.5 years.

**Table 1:** Participant characteristics.

	HIV-uninfected (n = 780)	HIV-infected (n = 894)	*P* value
Age, yr; median (IQR)	61.7 (55.0, 68.0)	55.5 (47.8, 62.2)	<.001
Age category n (%)			
18–45 yr	86 (11.0)	175 (19.6)	<.001
45–54 yr	109 (14.0)	256 (28.6)	
55–64 yr	300 (36.5)	317 (35.5)	
≥65 yr	285 (38.5)	146 (16.3)	
Race; n (%)			<.001
Caucasian	570 (73.1)	461 (51.6)	
African American	139 (17.8)	273 (30.5)	
Hispanic /Other	71 (9.1)	160 (17.9)	
Enrolled after 2001 n (%)	260 (33.5)	545 (61.0)	<.001
Educational level (below 12 yr) n (%)	25 (3.2)	63 (7.1)	<.001
Cumulative pack-year of smoking: median (IQR)	0.3 (0, 19.4)	2.4 (0, 19.6)	.040
Alcohol use >13 drinks per week n (%)	77 (9.9)	67 (7.4)	.016
Body mass index, kg/m^2^; median (IQR)	26.7 (24.1, 30.1)	26.0 (23.2, 29.2)	<.001
Systolic blood pressure, mm Hg; median (IQR)	131 (121, 141)	128 (118, 138)	<.001
On hypertension medication; n (%)	309 (39.8)	321 (37.1)	.26
On diabetes medication; n (%)	79 (10.2)	97 (10.9)	.65
On cholesterol lowering medication; n (%)	294 (37.6)	331 (36.8)	.74
Hepatitis C virus infection; n (%)	28 (3.6)	55 (6.2)	<.001
Fasting glucose, mg/dL; median (IQR)	93 (85, 101)	93 (85, 103)	.71
Total cholesterol, mg/dL; median (IQR)	178 (152, 204)	176 (151, 201)	.21
HDL cholesterol, mg/dL; median (IQR)	51.8 (43.6, 61.7)	48.2 (41.1, 57.0)	<.001
Cocaine use since last visit n (%)	47 (6.1)	95 (10.8)	.001
HIV disease severity factors			
HIV RNA viral load <20 copies/mL		724 (81.0)	
CD4+ T cell count (cells/mm^3^); median (IQR)		681 (501, 887)	
Nadir CD4+ T cell count (cells/mm^3^); median (IQR)		322 (206, 456)	
On cART n (%)		809 (91.3)	
Duration of cART, yr; median (IQR)		12.5 (5.0, 16.8)	
History of clinical AIDS n (%)		88 (9.8)	
Resting ECG only	179 (22.9)	214 (23.9)	.61
Ambulatory ECG only	22 (2.8)	19 (2.1)	
Resting ECG and ambulatory ECG	579 (74.2)	661 (73.9)	
Atrial fibrillation or atrial flutter n (%)	26 (3.3)	12 (1.3)	.006

AIDS = acquired immunodeficiency syndrome, cART = combination antiretroviral therapy, ECG = electrocardiogram, HDL = high density lipoprotein, HIV = human immunodeficiency virus, IQR = interquartile range, RNA = ribonucleic acid.

The majority of the sample, 1240 men (74%), completed both ambulatory and resting ECG monitoring; 393 men (23%) had only resting ECG, and 41 men (2%) had only ambulatory ECG monitoring. Participants wore the Zio XT ambulatory ECG for a median of 13.0 days (interquartile range 5.9, 14.0). Among the participants who had ambulatory ECG recordings, 54 participants had <25% analyzable time (relative to wear time) and/or <24 hours of analyzable time on ambulatory ECG; therefore, their ambulatory ECG results were excluded from the analyses. These 54 participants all had technically adequate resting 12 lead ECG data. For this analysis, 1186 (71%) participants contributed both ambulatory ECG and resting ECG results, 447 (27%) participants contributed only resting ECG and 41 (2%) participants contributed only ambulatory ECG results. The overall prevalence of AF was 2.3% (38 cases). There were 36 cases of AF, 1 case of atrial flutter, and 1 case of both AF and atrial flutter. Among the 33 men with AF on ambulatory ECG monitoring, 55% (n = 18) had a low burden of AF of <5% time in AF, while 18% (n = 6) had 5% to 80% time in AF, and 27% (n = 9) were in persistent AF (100% time in AF) for the duration of the Zio XT recording.

The prevalence of AF in white men was 3.6% (37/1031). There was only one case of AF among black men (1/412 = 0.24%) and no cases among the 231 Hispanic men and men of other races. There were 26 cases of AF among the 780 HIV– men and 12 cases among the 894 HIV+ men. The age and race standardized prevalence of AF was 3.3% (95% CI: 2.1–4.6) among HIV– men and 3.0% (95% CI: 1.1–5.0) among HIV+ men. Associations between HIV serostatus, cardiovascular risk factors, and the odds of AF are shown in Table 2. There was no AF detected among participants with recent cocaine use, resulting in exclusion of this variable from the fully adjusted model. In unadjusted analyses, the odds of AF were 61% lower among HIV+ compared with HIV– men (OR 0.39, 95% CI 0.20–0.79: *P* < .001). However, after adjusting for age and race, we found no difference in the odds of AF by HIV serostatus (OR 0.77, 95% CI 0.37–1.58; *P* = 0.47). After further adjustment for cardiovascular risk factors, there was no difference between the odds of AF comparing HIV+ to HIV– men (OR 0.84, 95% CI 0.39–1.81; *P* = .66). The odds of AF were 8% higher for each yearly increase in age (OR 1.08, 95% CI 1.03–1.13; *P* = .002) and the odds of AF comparing non-Caucasians to Caucasians was 0.06 (95% CI 0.01–0.53, *P* = .01). There was an increase in the odds of AF with higher fasting blood glucose (OR 1.01 95% CI 1.00–1.02; *P* = .01) and among participants who were on blood pressure medication (OR 2.61 95% CI 1.18–5.76, *P* = .02), but no other significant associations with cardiovascular risk factors were noted. Among HIV+ men, the median CD4+ T cell counts were similar among men with AF compared with those without AF (722 cells/mm^3^ vs 680 cells/mm^3^, age adjusted *P* value = .30). Among the men living with HIV 92% (11/12) of those with AF were on cART and all these men had undetectable viral load. Similar results were seen when analyses only included ambulatory ECG results (data not shown).

**Table 2:** Associations between HIV serostatus and atrial fibrillation.

	Model 1 unadjusted odds ratio (95% CI), *P*-value n = 1674	Model 2 adjusted odds ratio (95% CI), *P*-value n = 1674	Model 3 adjusted odds ratio (95% CI), *P*-value n = 1587
HIV-infected (vs HIV-uninfected)	**0.39 (0.20–0.79) *P* = .008**	0.77 (0.37–1.58) *P* = .47	0. 84 (0. 39–1.81) *P* = .66
Age per year	**1.10 (1.06–1.14) *P* < .001**	**1.08 (1.03–1.12) *P* < .001**	**1.08 (1.02–1.13) *P* = .002**
Race category			
Caucasian	Reference	Reference	Reference
Non-Caucasian	**0.04 (0.01–0.31) *P* = .002**	**0.08 (0.01–0.66) *P* = .02**	**0.06 (0.01–0.53) *P* = .01**
Body mass index (per 1 kg/m^2^)			1.02 (0.96–1.09) *P* = .46
Alcohol use >13 drinks per week (vs ≤13)			0.73 (0.24–2.40) *P* = .55
Cumulative pack-years of smoking			0.99 (0.98–1.01) *P* = .23
Systolic blood pressure (per 1 mm Hg)			1.00 (0.98–1.02) *P* = .94
Fasting glucose (per 1 mg/dL)			**1.01 (1.00–1.02) *P* = .01**
On hypertension medications (vs none)			**2.61 (1.18–5.76) *P* = .02**
On diabetes medications (vs none)			0.63 (0.21–1.90) *P* = .41

HIV = human immunodeficiency virus.

## 4 Discussion

We found no difference in the frequency of AF between HIV+ and HIV– men, after adjusting for demographics or additionally adjusting for cardiovascular risk factors. As expected, AF was strongly associated with aging. There was generally a low prevalence of AF in this cohort and a remarkably low prevalence of AF in non-Caucasian men. Among HIV+ participants, there was no association between AF and CD4+ T cell counts.

Our findings are similar to a matched case-control hospital-based study which found higher odds of AF in HIV+ participants compared with HIV– controls, however, after adjusting for confounders, the findings in that study also became null.^[12]^ Our unadjusted results suggested that HIV+ men had a lower prevalence of AF than HIV– men, contrary to the hypothesis. These results appear to be secondary to the differences in age and race/ethnicity between the groups, and were further attenuated in the fully adjusted model. Previous studies have also demonstrated a lower incidence of AF in African-American compared with Caucasian individuals, however, the differences in our study population are more marked than reported previously.^[17–19]^ Sardana et al^[13]^ demonstrated a higher risk of AF among HIV+ individuals, however, the disparity in our findings may be due to key differences in the study population, mode of outcome ascertainment, and study design.

People living with HIV are at increased risk for CVD, including coronary artery disease and heart failure, especially among those with prior exposure to uncontrolled viremia with resultant inflammation and immunodeficiency.^[5]^ We previously demonstrated that HIV+ men in MACS had a longer QT interval on resting ECG than HIV– men, which was associated with elevated inflammatory marker levels.^[15]^ Studies also suggest an increased risk for sudden cardiac death in HIV+ individuals.^[20]^ Although people living with HIV have a greater risk for CVD, the results of our study and others suggest that HIV is not associated with AF, a potent risk factor for stroke.^[12]^

### 4.1 Strengths and limitations

There are some limitations to our study, which included only men. Conclusions cannot be drawn about women, who were not represented. As with any observational study, we cannot rule out the possibility of residual confounding. The prevalence of AF among HIV+ men was low, which limited the power to assess multivariable adjusted associations between AF with HIV specific factors, such as CD4+ T cell counts, ART, and HIV viral load.

There are many strengths, including the relatively large sample size, inclusion of an HIV uninfected comparison group, detailed information on CVD risk factors, and similarities in behaviors of HIV+ and at-risk HIV-uninfected participants. Most importantly, compared to prior studies, the ascertainment of AF using resting ECG and ambulatory ECG monitoring at a research study visit allows for a diagnostic assessment not affected by biases related to the frequency of medical visits, as might be seen in HIV+ individuals.

#### 4.1.1 Future directions and implications for clinical practice

Our study included ambulatory ECG monitoring as a research examination. Future studies are needed to determine clinical indications for ambulatory ECG monitoring in individuals at risk for AF, to identify those who might benefit from anticoagulation to reduce risk for thromboembolic events.

## 5 Conclusion

We found no association between HIV and AF in this cohort in which viral replication among the HIV+ men is generally suppressed. The overall prevalence of AF was low and was rare in African-American men.

## Acknowledgments

Data in this manuscript were collected by the Multicenter AIDS Cohort Study (MACS), now the MACS/WIHS Combined Cohort Study (MWCCS). The contents of this publication are solely the responsibility of the authors and do not represent the official views of the National Institutes of Health (NIH). MWCCS (Principal Investigators): Baltimore CRS (Todd Brown and Joseph Margolick), U01-HL146201; Data Analysis and Coordination Center (Gypsyamber D'Souza, Stephen Gange and Elizabeth Golub), U01-HL146193; Chicago-Northwestern CRS (Steven Wolinsky), U01-HL146240; Los Angeles CRS (Roger Detels), U01-HL146333; Pittsburgh CRS (Jeremy Martinson and Charles Rinaldo), U01-HL146208. The MWCCS is funded primarily by the National Heart, Lung, and Blood Institute (NHLBI), with additional co-funding from the Eunice Kennedy Shriver National Institute Of Child Health & Human Development (NICHD), National Institute On Aging (NIA), National Institute Of Dental & Craniofacial Research (NIDCR), National Institute Of Allergy And Infectious Diseases (NIAID), National Institute Of Neurological Disorders And Stroke (NINDS), National Institute Of Mental Health (NIMH), National Institute On Drug Abuse (NIDA), National Institute Of Nursing Research (NINR), National Cancer Institute (NCI), National Institute on Alcohol Abuse and Alcoholism (NIAAA), National Institute on Deafness and Other Communication Disorders (NIDCD), National Institute of Diabetes and Digestive and Kidney Diseases (NIDDK), National Institute on Minority Health and Health Disparities (NIMHD), and in coordination and alignment with the research priorities of the National Institutes of Health, Office of AIDS Research (OAR).

## Author contributions

**Conceptualization:** Sabina A. Haberlen, Todd T. Brown, Matthew J. Feinstein, Mallory D. Witt, Jared W. Magnani, Elsayed Z. Soliman, Katherine C. Wu, Wendy S. Post.

**Formal analysis:** Ngozi C. Osuji, Sabina A. Haberlen.

**Methodology:** Ngozi C. Osuji, Wendy S. Post.

**Project administration:** Wendy S. Post.

**Software:** Ngozi C. Osuji, Sabina A. Haberlen.

**Supervision:** Wendy S. Post.

**Validation:** Wendy S. Post.

**Writing – original draft:** Ngozi C. Osuji, Sabina A. Haberlen, Wendy S. Post.

**Writing – review & editing:** Ngozi C. Osuji, Sabina A. Haberlen, Hiroshi Ashikaga, Todd T. Brown, Matthew J. Feinstein, Mallory D. Witt, Jared W. Magnani, Elsayed Z. Soliman, Katherine C. Wu, Wendy S. Post.
